# Adipose-Derived Stem Cells Ameliorate Allergic Airway Inflammation by Inducing Regulatory T Cells in a Mouse Model of Asthma

**DOI:** 10.1155/2014/436476

**Published:** 2014-08-26

**Authors:** Kyu-Sup Cho, Mi-Kyung Park, Shin-Ae Kang, Hee-Young Park, Sung-Lyong Hong, Hye-Kyung Park, Hak-Sun Yu, Hwan-Jung Roh

**Affiliations:** ^1^Department of Otorhinolaryngology and Biomedical Research Institute, Pusan National University Hospital, Busan 602-739, Republic of Korea; ^2^Department of Parasitology, Pusan National University School of Medicine, Yangsan 626-870, Republic of Korea; ^3^Department of Internal Medicine, Pusan National University Hospital, Busan 602-739, Republic of Korea; ^4^Department of Otorhinolaryngology and Research Institute for Convergence of Biomedical Science and Technology, Pusan National University Yangsan Hospital, Beom-eo li, Mul-geum eup, Yang-san si, Gyeongsangnam-do, Yangsan 626-770, Republic of Korea

## Abstract

Although several studies have demonstrated that mesenchymal stem cells derived from adipose tissue (ASCs) can ameliorate allergic airway inflammation, the immunomodulatory mechanism of ASCs remains unclear. In this study, we investigated whether regulatory T cells (Tregs) induction is a potential mechanism in immunomodulatory effects of ASCs on allergic airway disease and how these induced Tregs orchestrate allergic inflammation. Intravenous administration of ASCs significantly reduced allergic symptoms and inhibited eosinophilic inflammation. Airway hyperresponsiveness, total immune cell and eosinophils in the bronchoalveolar lavage fluid, mucus production, and serum allergen-specific IgE and IgG1 were significantly reduced after ASCs administration. ASCs significantly inhibited Th2 cytokines (IL-4, IL-5, and IL-13) and enhanced Th1 cytokine (IFN-*γ*) and regulatory cytokines (IL-10 and TGF-*β*) in the bronchoalveolar lavage fluid and lung draining lymph nodes. Furthermore, levels of IDO, TGF-*β*, and PGE_2_ were significantly increased after ASCs administration. Interestingly, this upregulation was accompanied by increased Treg populations. In conclusion, ASCs ameliorated allergic airway inflammation and improved lung function through the induction of Treg expansion. The induction of Treg by ASCs involves the secretion of soluble factors such as IDO, TGF-*β*, and PGE_2_ and Treg might be involved in the downregulation of Th2 cytokines and upregulation of Th1 cytokines production.

## 1. Introduction

Allergic rhinitis (AR) and asthma are chronic, reversible allergic airway diseases that have become a significant global public health concern [1]. Allergic airway diseases are characterized by Th2-skewed eosinophilic inflammation, mucus hypersecretion, and airway hyperresponsiveness (AHR) [1, 2]. The excessive activation of Th2 cells is thought to play a major role in allergic immune reaction, initiating and propagating inflammation through release of a number of Th2 cytokines, such as IL-4 and IL-13, that regulate isotype switching to allergen-specific IgE or IL-5, which recruits and activates eosinophils [3].

Mesenchymal stem cells (MSCs) represent an important stem cell population with multipotent capabilities which may have high utility for translational clinical applications. MSCs were initially isolated from bone marrow (BM) but are now shown to reside in almost adult organ and tissues [4]. Because of their capacity for differentiation, MSCs have emerged as a promising source for therapeutic applications in tissue engineering and regenerative medicine [5, 6]. In addition to their multilineage potential, MSCs derived from adipose tissue (ASCs) may share with other MSCs the unique ability to suppress immune responses and modulate inflammation [7]. MSCs can inhibit natural killer cell function [8, 9], modulate dendritic cell maturation [10], and suppress the allogeneic T cell response [8] by altering the cytokine secretion profile of dendritic cells and T cells induced by an allogeneic immune reaction. Although the ability of MSCs to modulate immune systems has led to increasing interest in using MSCs as a potential therapeutic modality for allergic airway diseases, only several studies demonstrated MSCs can ameliorate allergic airway inflammatory diseases, including asthma [11–13] and AR [14–17]. Moreover, the immunomodulatory mechanism of MSCs in allergic airway disease is not completely understood.

In this study, we evaluated the effects of MSCs on allergic inflammation, changes of regulatory T cells (Tregs), and cytokines in ovalbumin (OVA) induced asthmatic mouse model. Furthermore, we investigated whether Tregs induction is a potential mechanism in immunomodulatory effects of MSCs on allergic airway disease and how these induced Tregs orchestrate allergic inflammation.

## 2. Materials and Methods

### 2.1. Animals

Five-week-old female C57BL/6 mice were purchased from Samtako Co. (Osan, Republic of Korea, http://www.samtako.com.ipaddress.com/) and bred in a specific pathogen free animal facility. The animal study protocol was approved by the Institutional Animal Care and Use Committee of the Pusan National University School of Medicine.

### 2.2. Isolation and Culture of ASCs

Among MSCs, ASCs were used because of their abundance, relatively easy harvesting, and high proliferation potential. Adipose tissue was obtained from the abdominal fat of C57BL/6 mice. To isolate homogenous ASCs, adipose tissue was washed extensively with equal volumes of phosphate-buffered saline (PBS) and digested with 0.075% collagenase type I (Sigma, St. Louis, MO) at 37°C for 30 minutes. Enzyme activity was neutralized with α-modified Eagle's medium (α-MEM) containing 10% fetal bovine serum (FBS) and the sample was centrifuged at 1,200 ×g for 10 minutes to obtain a pellet. The pellet was filtered through a 100 *μ*m nylon mesh to remove cellular debris and incubated overnight at 37°C in 5% CO_2_ in control medium (α-MEM, 10% FBS, 100 unit/mL penicillin, 100 *μ*g/mL streptomycin). Following incubation, the plates were washed extensively with PBS to remove residual nonadherent red blood cells. The resulting cell population was maintained at 37°C in 5% CO_2_ in control medium. One week later, when the monolayer of adherent cells had reached confluence, cells were trypsinized (0.05% trypsin-EDTA; Sigma), resuspended in α-MEM containing 10% FBS, and subcultured at the concentration of 2,000 cells/cm^3^. For the experiments, we used the third or fourth passage of ASCs.

Flow cytometric analysis was used to characterize the phenotypes of the ASCs. At least 50,000 cells (in 100 *μ*L PBS, 0.5% bovine serum albumin (BSA), 2 mmol/l EDTA) were incubated with fluorescein isothiocyanate-labeled monoclonal antibodies against mouse stem cell antigen-1 (Sca-1), CD44, CD90, CD45, CD 117, and CD11b (BD Biosciences Clontech, Palo Alto, CA) or with the respective isotype control. After washing, labeled cells were analyzed by flow cytometry using FACSCalibur flow cytometer and the Cell Quest Pro software (BD Biosciences, San Diego, CA).

### 2.3. Mouse Model of Allergic Airways Inflammation

**Figure 1:**
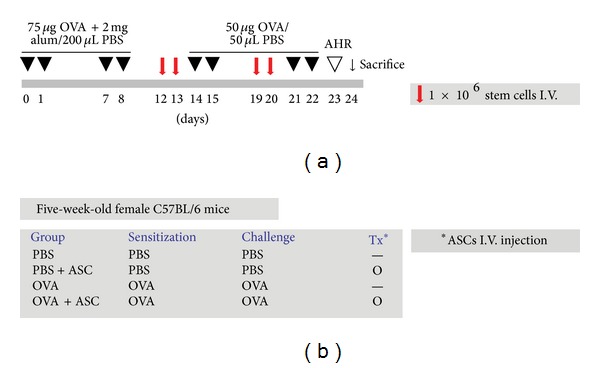
The experimental protocol and group. (a) The mice were sensitized on days 0, 1, 7, and 8 by intraperitoneal injection of OVA and challenged intranasally on days 14, 15, 21, and 22. 1 × 10^6^ purified ASCs were injected via the tail vein on days 12, 13, 19, and 20. (b) The mice were divided into four different groups in accordance with the different sensitization, challenge, and injection.

A mouse model of allergic airways inflammation was induced as previously reported with minor modification [18]. Briefly, mice were sensitized by intraperitoneal injection of 75 *μ*g of OVA (Sigma, St. Louis, MO, http://www.sigmaaldrich.com) in 2 mg of aluminum hydroxide (Sigma) in 200 *μ*L PBS on days 0, 1, 7, and 8. On days 14, 15, 21, and 22 after the initial sensitization, the mice were challenged intranasally with 50 *μ*g of OVA in 50 *μ*L PBS (Figure 1(a)).

### 2.4. Intravenous Transplantation of ASCs

ASCs were washed with PBS and suspended in PBS at a concentration of 1 × 10^7^ cells/mL. To evaluate the effect of ASCs, 0.1 mL of purified stem cells was injected with a 26-gauge needle via the mouse tail vein once a day on days 12, 13, 19, and 20 (Figure 1(a)).

Mice were divided into four groups, with five mice in each group: (a) PBS group mice were sensitized, pretreated, and challenged with PBS; (b) PBS+ASC group mice were sensitized and challenged with PBS but pretreated with ASCs; (c) OVA group mice were sensitized with OVA, pretreated with PBS, and then challenged with OVA; (d) OVA+ASC group mice were sensitized with OVA, pretreated with ASCs, and then challenged with OVA (Figure 1(b)). These experiments were performed four times according to the same protocol.

### 2.5. Evaluation of Nasal Symptoms

The frequency of sneezing and nasal rubbing that occurred in the 10-minute time period after the last OVA challenge was determined in a blind manner by two observers.

### 2.6. Measurement of Methacholine AHR

Twenty-four hours after the last challenge, the AHR was assessed in conscious, unrestrained mice using noninvasive whole-body plethysmography (Allmedicus, Seoul, Republic of Korea) as previously described [19]. In brief, the mice were placed in the plethysmography chamber and exposed to increasing concentrations of aerosolized methacholine at 0, 12.5, 25, and 50 mg/mL for 10 min. The enhanced pause (Penh) was calculated automatically based on the mean pressure generated in the plethysmography chamber during inspiration and expiration combined with the time of each phase. The Penh values calculated during each 3-minute interval were then averaged.

### 2.7. Differential Cell Counting in Bronchoalveolar Lavage Fluid

To obtain bronchoalveolar lavage fluid (BALF), the tracheas of the anesthetized mice were exposed and cut just below the larynx. A polyurethane flexible tube (0.4 mm in outer diameter, 4 cm in length, and attached to a blunt 24-gauge needle (Boin Medical Co., Seoul, Republic of Korea)) was placed into the trachea, after which the lung was lavaged once with the 800 mL of sterile warm PBS. The BALF samples were centrifuged for 5 min at 1,500 rpm at 4°C. The supernatants were then decanted and immediately frozen at −70°C. Cell pellets were resuspended and washed twice in PBS. The total cell numbers were counted using a hematocytometer. BALF cell smears were prepared using cytospin apparatus and stained with Diff-Quik solution (Sysmex Co., Kobe, Japan) to determine the differential cells counts in accordance with conventional morphological criteria. At least 500 cells per slide were evaluated in order to obtain the differential leukocyte counts.

### 2.8. Lung Histology and Inflammation Scoring

Lung tissues were removed after the lavage, fixed in 10% neutral formalin for 36 hours, and embedded in paraffin. The thin sections of the embedding tissues were stained with hematoxylin and eosin (H&E) and periodic acid-Schiff (PAS) for the identification of eosinophils and counting mucin-secreting cells, respectively. Lung inflammation was assessed by the degree of peribronchial and perivascular inflammation, which were evaluated on a subjective scale of 0–4 as previously described [20, 21]. The values were given according to the following inflammatory parameters: 0 when no inflammation was detectable, 1 was for occasional cuffing with inflammatory cells, 2 when most bronchi or vessels were surrounded by the depth of one to three cells, 3 when most bronchi or vessels were surrounded by the depth of four to five cells, and 4 when most bronchi or vessels were surrounded by the depth of more than five cells. For quantifying the goblet cell hyperplasia, the percentage of PAS-positive cells in epithelial areas was examined from 8 to 10 tissue sections per mouse.

### 2.9. Quantitative Real-Time PCR for IDO and TGF-*β*

RNA was extracted from the lung by using 1 mL of QIAzol (Qiagen science, Valencia, CA) and RNA extraction was conducted in accordance with the manufacturer's protocols, transcribing 2 *μ*g of RNA using moloney murine leukemia virus (M-MLV) reverse transcriptase (Promega, Madison, WI). Indoleamine 2,3-dioxygenase (IDO) (forward, 5′-GATGAAGAAGTGGGCTTTGC-3′; reverse, 5′-TCCAGTTTGCCAAGACACAG-3′) and TGF-*β* (forward, 5′-CTACCTTTCCTTGGGAGACC-3′; reverse, 5′-CGGGAGTGGGAGCAGAA-3′) RNA levels were quantified, relative to housekeeping gene, GAPDH, using iCycler (Bio-Rad laboratories Inc., Hercules, CA) real-time PCR machines following manufacturer's instructions. The relative expression of the gene was then calculated as the ratio to a housekeeping gene using the gene-x program (Bio-Rad laboratories Inc.).

### 2.10. Measurement of Serum Immunoglobulin and PGE_2_

At 48 hours after last OVA challenge, serum was collected from mice via cardiac puncture. Total and OVA-specific immunoglobulins (Ig E, IgG1, and IgG2a) and PGE_2_ were determined by enzyme-linked immunosorbent assay (ELISA). All of these were conducted in accordance with the manufacturer's instructions (R&D Systems, Minneapolis, MN). Absorbance (450 nm) was measured with an ELISA plate reader (Molecular Devices, Sunnyvale, CA).

### 2.11. Expression of Cytokines in the BALF and Lung Draining Lymph Nodes

The concentration of mouse IL-4, IL-5, IL-10, IL-13, interferon- (IFN-) *γ*, and transforming growth factor- (TGF-) *β* expression in the BALF and in the stimulated supernatants of lung draining lymph nodes (LLNs) was examined using commercially available ELISA kits in accordance with the manufacturer's instructions (eBioscience, San Diego, CA). The absorbance of the final reactant was determined at 450 nm with an ELISA plate reader (Molecular Devices).

### 2.12. Determination of Tregs and Intracellular Cytokine Staining

To evaluate the recruitment of T_reg_ induced by ASCs treatment, the LLN cells were cultured in plate-coated anti-CD3 for 3 hours from the LLNs of OVA-induced asthmatic mice and ASC-treated asthmatic mice. The cells were stained with anti-CD25-APC (0.2 mg/mL), anti-CD4-FITC (0.5 mg/mL), and anti-Foxp3 (0.2 mg/mL) in accordance with the manufacturer's recommendations (BD Biosciences, San Jose, CA).

To stain intracellular IFN-*γ* and IL-4, the LLN cells were first stained for CD4, subsequently fixed, permeabilized using Cytofix/Cytoperm Kit (BD Biosciences), and incubated with PE-cy7-conjugated anti-IFN-*γ* or PE-conjugated anti-IL-4. Fluorescence was measured using a FACS CantoII cytometer (BD Biosciences) equipped with Canto software (BD Biosciences).

### 2.13. Statistical Analysis

All experiments were repeated a minimum of three times. Data are expressed as mean ± SEM. Statistical significance was assessed by the Student's *t*-test or ANOVA using the SPSS software package version 18.0 (SPSS Inc., Chicago, IL). A value of *P* < 0.05 was considered significant.

## 3. Results

### 3.1. Isolation, Immunophenotypic Analysis, and Multilineage Differentiation of ASCs

**Figure 2:**
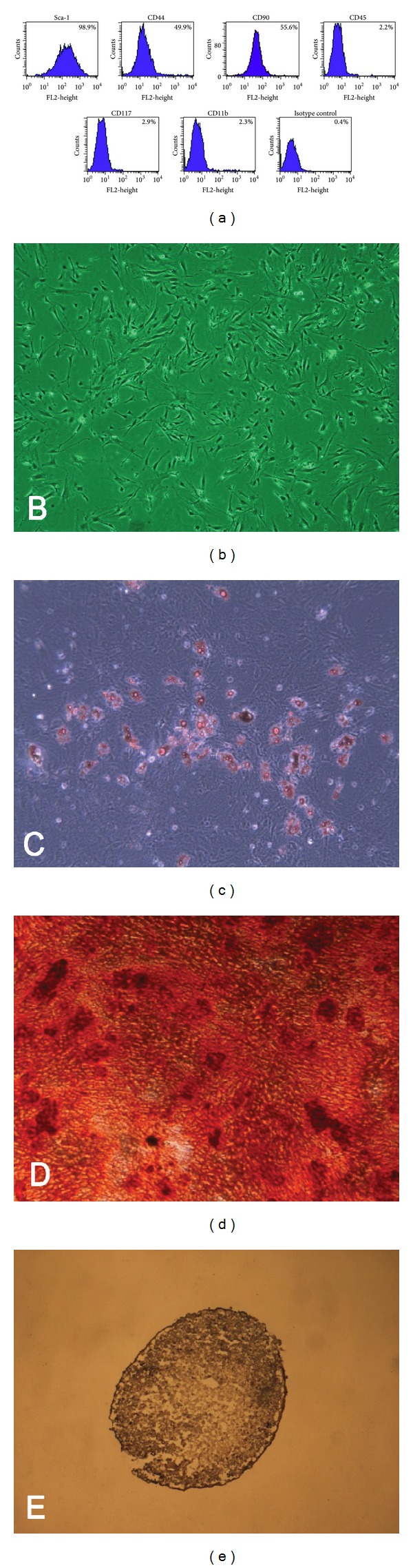
Characteristics of adipose-derived stem cells (ASCs). ASCs show characteristics of mesenchymal stem cells in the immunophenotypic analysis (a), fibroblast-like morphology (b), adipogenesis (c), osteogenesis (d), and chondrogenesis (e) (original magnification ×40).

The cultured ASCs from adipose tissue of C57BL/6 mice were negative for CD45, CD117, and CD11b but were positive for Sca-1, CD44, and CD90 (Figure 2(a)). These putative ASCs had a spindle shaped fibroblast-like appearance, similar to previously reported adipose tissue and bone marrow-derived MSCs (Figure 2(b)). The multilineage capacity of ASCs was demonstrated by incubating the cells in the media that promoted differentiation into the adipogenic, osteogenic, and chondrogenic lineage (Figures 2(c), 2(d), and 2(e)).

### 3.2. Systemic Administration of ASCs Suppresses Allergic Nasal Symptoms

To investigate whether the administration of ASCs inhibits the occurrence of nasal symptoms, clinical parameters were measured. The frequency of sneezing and nasal rubbing was significantly increased by OVA challenge. The number of nasal symptoms after the final challenge was significantly higher in the OVA group than in the PBS group (*P* < 0.001). Interestingly, ASCs treatment before the challenge phase significantly reduced the number of nasal symptoms (*P* = 0.023) (data not shown).

### 3.3. Systemic Administration of ASCs Reduces AHR, Lung Inflammation, and Mucus Production

**Figure 3:**
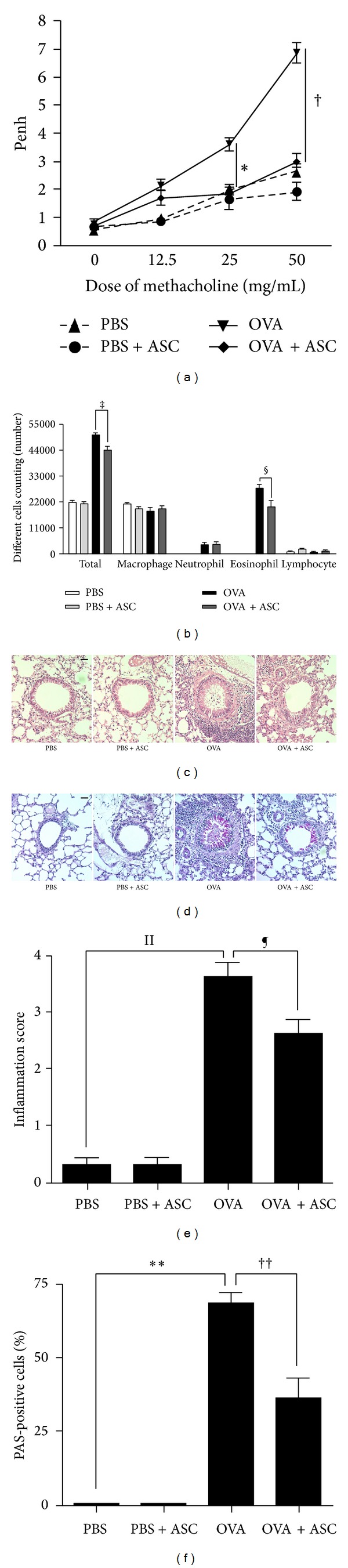
Effect of adipose-derived stem cells (ASCs) on AHR, lung inflammation, and mucus production. Airway reactivity to methacholine challenge (a) and the number of total inflammatory cells and eosinophils (b) was significantly decreased in the OVA+ASC group compared to the OVA group. (c) ASCs treatment decreased the infiltration of eosinophils ((c), (e)) and PAS-positive cells ((d), (f)) around the airway and blood vessel (H&E, PAS ×200).

To identify the effect of ASCs on lung function, AHR was measured. Penh values in four groups were increased with increasing concentrations of methacholine. Penh values in asthmatic mice at 25–50 mg/mL were significantly higher than those in the PBS and OVA+ASC group. ASCs treatment significantly decreased at different concentrations from 25 to 50 mg/mL in response to methacholine in asthmatic mice (*P* = 0.002 and *P* < 0.001, resp.) (Figure 3(a)).

To determine the effect of ASCs on inflammation in asthmatic mice, inflammatory cells in BALF were stained and counted. The number of total inflammatory cells and eosinophils was increased in the BALF of OVA group compared to PBS group. However, ASCs treatment significantly decreased the number of total inflammatory cells and eosinophils in asthmatic mice (*P* = 0.009 and *P* = 0.010, resp.) (Figure 3(b)).

No obvious infiltration of inflammatory cells was found in the PBS and PBS+ASC group, but a greater number of eosinophils around the peribronchial and perivascular area were seen in the OVA group (*P* < 0.001) (Figures 3(c) and 3(e)). Concurrently, PAS-stained goblet cell hyperplasia, as demonstrated by the increased number and size of goblet cells, also occurred within the respiratory epithelium in the OVA group (*P* < 0.001) (Figures 3(d) and 3(f)). However, this hyperplasia was not found in the airways in both the PBS group and PBS+ASC group. Interestingly, ASCs treatment induced a significant reduction in the number of eosinophils (*P* = 0.002) (Figures 3(c) and 3(e)) and goblet cell hyperplasia (*P* = 0.02) (Figures 3(d) and 3(f)) in asthmatic mice.

### 3.4. Systemic Administration of ASCs Decreases IgE and IgG1

**Figure 4:**
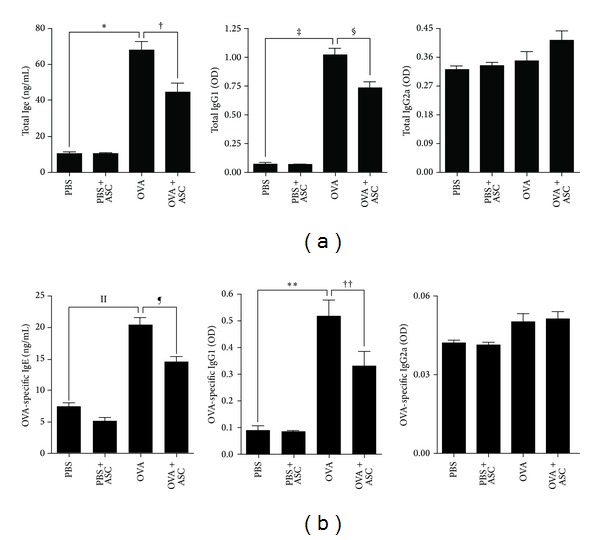
Effect of adipose-derived stem cells (ASCs) on serum levels of immunoglobulin. Total (a) and OVA-specific (b) IgE and IgG1 levels were significantly higher in the OVA group than in the PBS group. Systemic administration of ASCs resulted in a significant decrease in total (a) and OVA-specific (b) IgE and IgG1 levels in asthmatic mice.

To determine whether injected ASCs affect Th2-specific immunoglobulin concentrations in the serum, the total and OVA-specific IgE, IgG1, and IgG2a levels were determined. Total and OVA-specific IgE and IgG1 levels were significantly higher in the OVA group than in the PBS group (all *P* < 0.001). However, systemic administration of ASCs resulted in a significant decrease in total IgE and IgG1 (all *P* = 0.033) and OVA-specific IgE and IgG1 levels (*P* = 0.025 and *P* = 0.013, resp.) in asthmatic mice. There were no significant differences in serum total and OVA-specific IgG2a levels in all groups (Figure 4).

### 3.5. Systemic Administration of ASCs Alters Cytokine Levels in Both the BALF and the LLN

**Figure 5:**
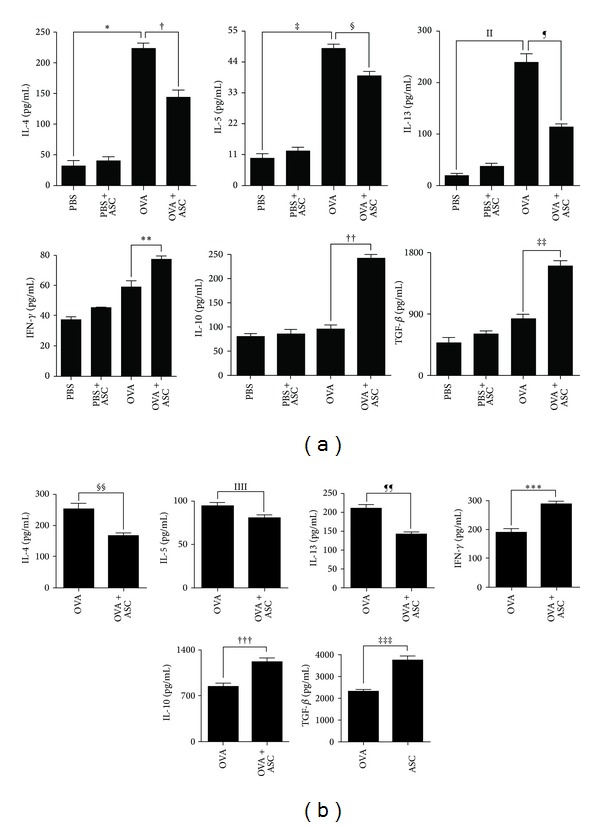
Effect of adipose-derived stem cells (ASCs) on cytokine levels of BALF (a) and LLNs (b). IL-4, IL-5, and IL-13 levels were significantly higher in the BALF of the OVA group than PBS group. ASCs treatment significantly decreased the levels of IL-4, IL-5, and IL-13 in the BALF and LLNs but increased the levels of IFN-*γ*, IL-10, and TGF-*β* in the BALF and LLNs.

To determine whether the administration of ASCs affects cytokine production, the cytokines in both the BALF and the LLN were analyzed. OVA-challenged mice showed significantly increased levels of IL-4, IL-5, and IL-13 in the BALF (all *P* < 0.001). However, ASCs treatment resulted in a significant decrease in IL-4, IL-5, and IL-13 in the BALF (*P* < 0.001, *P* = 0.020, and *P* < 0.001, resp.) and LLN (*P* < 0.004, *P* = 0.018, and *P* < 0.001, resp.). In contrast, higher levels of IFN-*γ* were observed in both the BALF and the LLN of OVA+ASC group compared to OVA group (*P* = 0.001 and *P* = 0.026, resp.). Interestingly, ASC treatment significantly increased IL-10 and TGF-*β* in the BALF (all *P* < 0.001) and LLN (all *P* = 0.001) of OVA+ASC group (Figure 5).

### 3.6. Systemic Administration of ASCs Enhances Tregs Expansion, Gene Expression of IDO and TGF-*β*, and PGE_2_

**Figure 6:**
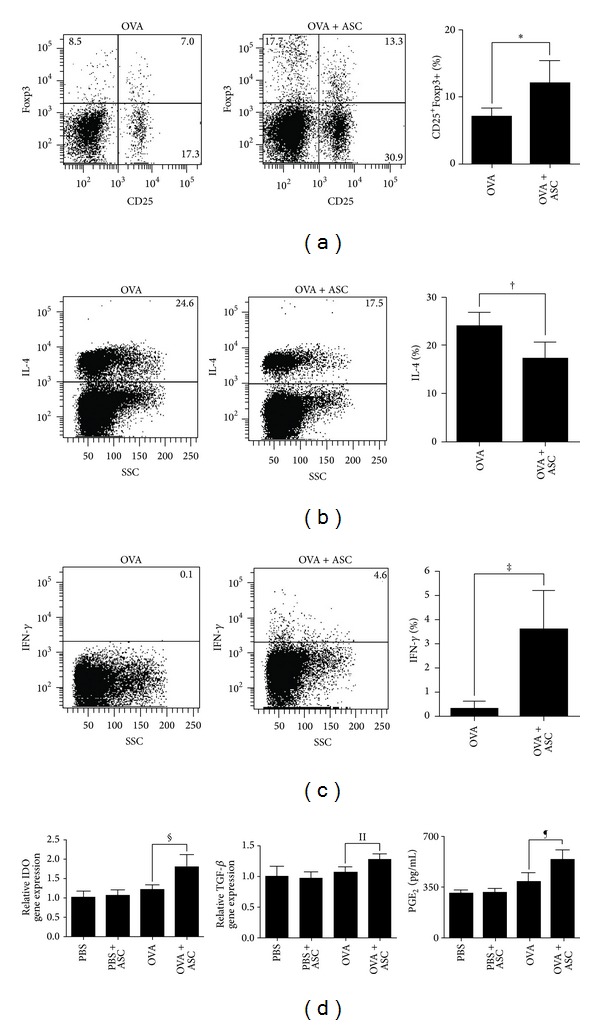
Effects of adipose-derived stem cells (ASCs) on Tregs, IL-4 or IFN-*γ* producing T cells, and gene expression of IDO and TGF-*β*. (a) ASCs treatment significantly increased the frequency of Tregs. IL-4^+^CD4^+^T cells (b) were significantly decreased, but IFN-*γ* ^+^CD4^+^T cells (c) were significantly increased by ASCs treatment. (d) The gene expression levels of IDO and TGF-*β* and PGE_2_ levels were significantly increased by ASCs treatment.

To study the mechanism underlying the immunomodulatory effects of ASCs in asthmatic mice, CD4^+^CD25^+^ Tregs, IL-4^+^CD4^+^ T cells, IFN-*γ* ^+^CD4^+^ T cells, and gene expression of IDO and TGF-*β* were explored. The population of Tregs in LLN of asthmatic mice was markedly increased by administration of ASCs in asthmatic mice (*P* = 0.017) (Figure 6(a)). IL-4^+^CD4^+^ T cells in LLNs were significantly decreased (*P* = 0.003) (Figure 6(b)) and IFN-*γ* ^+^CD4^+^ T cells in LLNs were significantly increased (*P* = 0.015) (Figure 6(c)) in the OVA+ASC group compared to the OVA group. In addition, gene expression levels of IDO and TGF-*β* of lung tissue and PGE_2_ levels in the serum were significantly increased in the OVA+ASC group compared to the OVA group (*P* = 0.003, *P* = 0.018, and *P* = 0.005, resp.) (Figure 6(d)).

## 4. Discussion

The immunomodulatory function of MSCs makes them promising candidates for allergic disease therapy. MSCs isolated from the BM and adipose tissues have similar morphology, phenotype, and differentiation capability [22]. Furthermore, both BM-MSCs and ASCs have immunosuppressive properties [23]. Because of their abundance, relatively easy harvesting, and high proliferation potential, ASCs might be a more useful source for cell therapy of allergic airway experimental studies [24]. Administration of MSCs can ameliorate severity of acute lung injury and fibrosis [25–27] and modulation of proinflammatory and anti-inflammatory cytokines is considered as the main beneficial effect of MSCs. Since asthma is characterized as chronic airway inflammation with eosinophilic infiltration and unbalance between Th1- and Th2-derived cytokines, we propose that ASCs-driven immunomodulation contributes to attenuation of airway inflammation in asthma, consequently improving lung function.

In this study, OVA challenge induced an infiltration of inflammatory cells in the airway and BALF as well as goblet cell hyperplasia in the airway of asthmatic mice. ASCs administration led to a significant histological and functional improvement in asthmatic mice. ASCs significantly decreased total cell number and eosinophils in BALF and improved lung pathology such as lung inflammation scores and goblet cell hyperplasia. These findings indicate that ASCs inhibited recruitment of eosinophils and mononuclear cells into the airway and BALF and reduced AR symptoms and AHR in asthmatic mice, which is consistent with previous studies [11, 12, 15, 17]. However the mechanisms underlying the beneficial effect of ASCs in allergic airway diseases are unclear.

As expected, ASCs administrations significantly decreased total and OVA-specific IgE and IgG1 serum levels. The levels of Th2 cytokines (IL-4, IL-5, and IL-13) were significantly decreased after the administration of ASCs, whereas IFN-*γ* and regulatory cytokines (IL-10 and TGF-*β*) were significantly increased in the BALF and LLN. We also demonstrated that the ratio of Tregs in ASCs-treated asthmatic mice was significantly higher than in untreated mice, which was similar to previous studies that indicate that BM-MSCs preferentially activate CD4+CD25+ T cell subsets, which are the main underlying mechanisms for immunosuppressive activity of MSCs [8, 28]. BM-MSCs prevented the occurrence of severe, irreversible damage to bone and cartilage in murine rheumatoid arthritis model by inducing production of antigen-specific Tregs [29]. However, the effect of MSCs on Tregs in allergic airway diseases is still unknown. Our study showed that ASCs administration significantly increased the ratio of Tregs in the LLNs of asthmatic mice. Furthermore, IL-4^+^CD4^+^ T cells were significantly reduced after ASCs treatment, whereas IFN-*γ* ^+^CD4^+^ T cells were significantly increased in the LLNs. Our findings, and those from other reports, provide evidence that Treg expansion plays a central role in the immunomodulatory properties of ASCs.

Tregs are a unique T cell population with strong immunosuppressive properties. CD4^+^CD25^+^ Tregs are impaired quantitatively and functionally and also play a protective role in suppressing airway eosinophilic inflammation and the development of airway hyperreactivity in asthma [30]. The induction of Tregs by MSCs involves not only direct contact between MSCs and CD4^+^ T cells, but also the secretion of soluble factors such as IDO, TGF-*β*, and PGE_2_ [31]. Treatment with IFN-*γ* causes MSCs to express the protein IDO and exhibit functional activity of IDO, which in turn degrades essential tryptophan and results in kynurenine synthesis and thereby suppresses lymphocyte proliferation [32]. Neutralizing antibodies against TGF-*β* and BM-MSCs derived from TGF-*β*1-KO mice eliminated the beneficial effect of MSCs, suggesting that the BM-MSCs-derived TGF-*β* is critical in suppressing the allergic responses [33, 34]. Coculturing T cells with MSCs resulted in elevated levels of PGE_2_, and treatment with inhibitors of PGE_2_ production mitigated the MSCs-mediated immune modulation [35, 36]. In this study, we observed that ASCs significantly increased IDO and TGF-*β* expression in lung tissue and PGE_2_ levels in the serum of OVA-sensitized mice, suggesting that IDO, TGF-*β*, and PGE_2_ might be the major soluble factors responsible for Treg expansion in LLNs.

**Figure 7:**
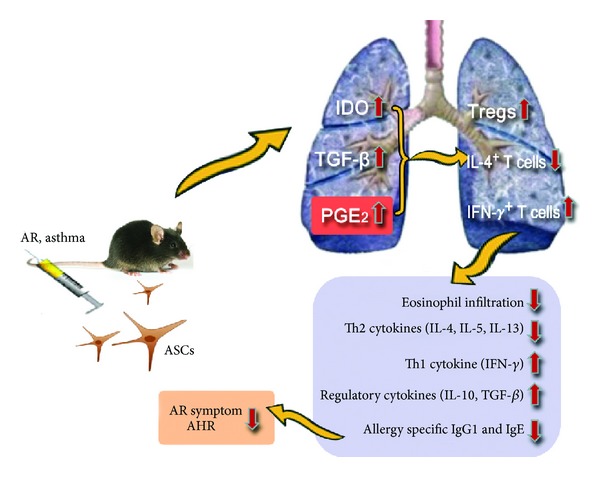
Schematic presentation of plausible mechanisms by which ASCs regulate the allergic airway diseases. ASCs migrated to the lung by intravenous administration secrete a variety of soluble factors including IDO, TGF-*β*, and PGE_2_. Through a soluble factor or direct contact of ASCs with T lymphocytes, ASCs initiate the expansion of Tregs. Tregs secrete IL-10 and TGF-*β* which ultimately lead to decrease of lung eosinophil infiltration, as well as allergy-specific Th2 cytokines and Ig production.

Our previous studies demonstrated that lungs are the primary site of ASCs accumulations following intravenous administration and developing allergic environment is capable of attracting and retaining more ASCs than unaffected lungs [11, 15]. Although it remains unclear whether systemic administration of ASCs became activated by which signaling mechanism, we present the supposed schematic drawing for the mechanism of ASCs effect on the basis of previous studies [11, 15, 32–36] and data of this study. ASCs migrated to the lung by intravenous administration secrete a variety of soluble factors including IDO, TGF-*β*, and PGE_2_. Through a soluble factor or direct contact of ASCs with T lymphocytes, ASCs induce the expansion of Tregs. Tregs secrete anti-inflammatory cytokines (IL-10, TGF-*β*), which ultimately lead to decrease of lung eosinophil infiltration, as well as allergy-specific Th2 cytokines and Ig production (Figure 7).

The present study demonstrates that intravenous treatment with ASCs in asthmatic mice provides a significant reduction of allergic airway inflammation and improvement of lung function. These immunomodulatory effects might be mediated by upregulating Tregs and partly involve increasing soluble factors such as IDO, TGF-*β*, and PGE_2_.
